# Reliability and validity evaluation of the appropriate antibiotic use self-efficacy scale for Chinese adults

**DOI:** 10.1186/s12889-022-13729-1

**Published:** 2022-07-14

**Authors:** Liying Wang, Chunguang Liang, Haitao Yu, Hui Zhang, Xiangru Yan

**Affiliations:** School of Nursing, Jinzhou Medical University, No 40, Section 3, Songpo Road, 121001 Jinzhou, China

**Keywords:** Appropriate antibiotic use, Antibiotic resistance, Self-medication, Antibiotics use self-efficacy, Medication self-efficacy

## Abstract

**Background:**

Antibiotic resistance is one of the greatest threats to global public health. Inappropriate use of antibiotics can lead to an increase in antibiotic resistance. Individual self-efficacy in the appropriate use of antibiotics plays a key role, especially in China where the population has easy access to antibiotics. However, there are no tools available to assess the self-efficacy of appropriate antibiotic use for Chinese adults. We aimed to translate and develop a Chinese version of the Appropriate Antibiotic Use Self-Efficacy Scale (AAUSES), and validate its reliability and validity.

**Methods:**

A total of 659 adults were recruited to participate in the questionnaire. The original version scale was first translated into Chinese using the backward and forward translation procedures. The internal consistency reliability of the scale was measured by the Cronbach alpha coefficient, the test-retest reliability, and the corrected item-total correlation. The validity of the scale was assessed by the content validity index, exploratory factor analysis, and confirmatory factor analysis.

**Results:**

The content validity index of the scale was 0.96. Exploratory factor analysis (EFA) supported a 4-factor structure of the translated questionnaire, and the discriminant validity of the scale was good. Confirmatory factor analysis (CFA) showed ﻿i﻿n the model fitness index, the chi-square degree of freedom was 2.940, the goodness-of-fit index(GFI) was 0.929, the incremental fit index (IFI) was 0.908, the comparative fit index(CFI) was 0.906, root mean square error of approximation(RMSEA) was 0.077, and standardized root mean residual (SRMR) was 0.0689, and the model fitting indexes were all in the acceptable range. Cronbach alpha coefficient for the scale was 0.910. The test-retest reliability was 0.947, and the corrected item-total correlations for the items ranged from 0.488 to 0.736. Self-efficacy for appropriate antibiotic use in adults varied by education, occupation, income, place of residence, and whether or not they had heard of antibiotic resistance.

**Conclusions:**

The results indicated that the Chinese version of the AAUSES had good reliability and validity. Therefore, it can be considered a tool to evaluate the appropriate antibiotic use self-efficacy of adults in China.

## Introduction

Antibiotics are among the most cost-effective and life-saving drugs, helping to extend the life expectancy of patients [1]. Researchers predicted that without dramatic changes, antibiotic consumption in 2030 could be 200% higher than in 2015 [2]. However, inappropriate and excessive use of antibiotics is a significant contributor to antibiotic resistance [3]. Antibiotic resistance has led to serious public health and economic consequences, with drug-resistant infections causing approximately 700,000 deaths globally each year. This number is expected to increase to 10 million by 2050, with associated costs of up to USD100 trillion globally if no action is taken [4]. Therefore, it is important to take action to combat antibiotic resistance.

The widespread inappropriate use of antibiotics by humans has accelerated the development of antibiotic resistance [5–7]. Globally, more than 50% of pharmacy customers buy antibiotics without a prescription, and this situation is even worse in developing countries [8, 9]. A review showed that the prevalence of antibiotic self-medication in South East Asia is around 50% [10], and approximately 43% of patients worldwide use antibiotics to treat respiratory infections [11]. Although antibiotics are prescribed, available research suggests that people’s behavior also plays a role in the irrational use of antibiotics [12–14], such as buying antibiotics over-the-counter, self-medicating with antibiotics, and storing and sharing antibiotics [15, 16] In addition, public behavior can also influence the rational use of antibiotics by doctors through expectations and pressure to use antibiotics, which is also seen as a key factor leading to unnecessary use of antibiotics by doctors [17, 18].

China is one of the countries that consume the most antibiotics and has one of the highest prevalence of antimicrobial resistance in the world [19, 20]. Excessive and irrational use of antibiotics has also been a concern in China. For example, more than half of all customers in China can obtain antibiotics without a prescription [21], which may further exacerbate antibiotic self-medication. And a considerable proportion of people cannot appropriately use antibiotics. The Chinese State Food and Drug Administration surveyed 7915 residents, 23.9% of whom said that when they had a cold, they would take antibiotics themselves rather than see a doctor [22]. Another study conducted in rural areas of China found that 46.3% of villagers experienced antibiotic self-medication [23]. It is estimated that about 58% of antibiotic misuse is due to irrational use of antibiotics by the general population, while doctors prescribe irrational antibiotics in 42% [24].

Existing studies have found that inappropriate antibiotic is associated with the following reseasons: antibiotic prescribing by non-infectious disease physicians [25], antibiotic dispensing in pharmacies [26, 27], and public knowledge, attitude and practice of antibiotic use [28]. The factors determining the appropriate use of antibiotics by individuals are influenced by several aspects, including consumers’ lack of knowledge about the appropriate use of antibiotics and their adverse effects [29–31], as well as their beliefs, expectations and personal experiences with antibiotics [32, 33]. Knowledge, attitudes and practice (KAP) studies are often a preferred method to achieve this [34–37]. These KAP studies focused on knowledge, attitudes and misconceptions about antibiotics and irrational behavior, but did not delve into the self-efficacy of individuals to use antibiotics rationally and were limited methodologically to disaggregated survey data.

Self-efficacy is one of the most powerful predictors of behavior change and disease self-management [38–40]. The concept of self-efficacy was first introduced by Bandura, an American psychologist, and is a core concept in Bandura’s social cognitive theory, self-efficacy is defined as the belief that one can successfully perform a behavior to achieve the desired outcome [41, 42]. Because self-efficacy beliefs work in conjunction with goals, outcome expectations, perceived environmental barriers and facilitators that regulate human motivation, behavior and well-being, the concept of self-efficacy has been used in pharmacotherapy practice [43–45], several medication self-efficacy scales also have been developed [46–49].

However, there is a lack of tools to measure the self-efficacy of rational antibiotic use. To assess this individual self-efficacy in the rational use of antibiotics, Erin M, Hill et al. first developed the Appropriate Antibiotic Use Self-Efficacy Scale (AAUSES). The AAUSES is a concise and validated instrument for measuring self-efficacy in the appropriate use of antibiotics [50]. At present, the scale is not used in other countries. Further confirmation is needed as to whether the AAUSES can be used directly to assess self-efficacy for rational antibiotic use in Chinese adults.

The study aimed to translate the original AAUSES translated into Chinese and further examine its reliability and validity among Chinese adults. Furthermore, we hypothesized that self-efficacy for rational antibiotic use was related to sociodemographic characteristics and clinical variables. Therefore, we compared the differences in the Chinese version of the AAUSES scores between different general data to validate our view.

## Methods

### Study design and participants

This study was a cross-sectional study and was conducted with a convenient sample of adults(age ≥ 18 years) from March to May 2021. Data was collected using Questionnaire Star, an online data collection platform in China. Two weeks later, 30 adults who participated in the first test were recruited to evaluate the test–retest reliability. The researchers examined the data and excluded questionnaires that had obvious logical errors and did not meet the criteria for this study (e.g., those < 18 years old). In this study, a total of 659 individuals took part in the survey. The survey was anonymous, but 30 of the participants who took part in the test were asked to write down their contact details so that the test–retest reliability could be assessed after two weeks. According to the guidelines for sample size, 5–10 participants per scale item would be sufficient to adequately test the validity and reliability of the scale [51]. The AAUSES has 13 items and the required sample size was calculated to be at least 65–130 participants. In this study, a total of 659 individuals took part in the survey. All participants are native Mandarin speakers and provided informed consent before participating in the study. The research procedures complied with the ethical standards of the Ethics Committee of Jinzhou Medical University (Grant Number:JZMULL2021009) as well as adhered to the ethical principles of the Helsinki declaration [52].

### Translation process

Before translation and validation, we obtained professor Hill EM’s permission. The forward–backward translation method according to the Brislin translation was used [53]. Firstly, The AAUSES has been independently translated into Chinese by one medical specialist and a psychologist. Secondly, the two experts and the researchers compared the translated Chinese versions of the questionnaire, discussed and corrected inconsistencies, and obtained a first draft of the Chinese version. Then, we invited two specialist in English who had not been exposed to the AAUSES to translate the first draft of the Chinese into English. Finally, the expert group compared and discussed the original scale, the first draft of the Chinese translation and the back-translated English scale. Changes were made to controversial items, focusing on linguistic and cultural adjustments to make the scale more appropriate for the Chinese context. A preliminary study was conducted with 10 adults. They were invited to complete the scale and were then asked about their understanding of the scale entries. They reported no difficulties in understanding and eventually developed the final Chinese version of the scale.

### Measurements

All participants completed the Chineses version of Appropriate Antibiotic Use Self-Efficacy Scale (AAUSES) [50] and the general self-efficacy scale (GSES) [54]. In addition, participants were asked to complete general profile information, including socio-demographic and clinical variables related to antibiotics. General data information included gender, age, education, place of residence, marital status and religion, occupation, availability of health insurance and monthly household income, and clinical variables include whether you have taken antibiotic, whether you have taken antibiotics to treat a cold or flu, the number of times you have used antibiotic to treat a cold or flu, whether you have heard of antibiotic resistance, the level of concern about resistance to antibiotic.

### Instruments

#### The Appropriate Antibiotic Use Self-Efficacy Scale (AAUSES)

The Appropriate Antibiotic Use Self-Efficacy Scale (AAUSES) was originally developed by Erin Hill et al., and is used to assess self-efficacy for appropriate antibiotic use in adults [50]. The scale consists of 13 items grouped into three subscales (minimization of antibiotics and trust in physician recommendations, avoidance of antibiotics for viral infections, and avoidance of taking old/ others’ antibiotics). The scale is evaluated on an 11-point scale. The total scores range from 0 (No confidence at all) to 100 (Totally confident), and higher scores indicate greater self-efficacy for appropriate antibiotic use. The original English version of AAUSES has shown good reliability and validity [50].

#### The General Self-Efficacy Scale (GSES)

The General self-efficacy scale (GSES) developed by Jerusalem and Schwarzer [54]. This scale measures an individual’s confidence in his or her ability to cope with a wide range of stressful or challenging demands. The GSES has been translated into Chinese, and the Chinese version of the GSES (C-GSES) has demonstrated good reliability with Cronbach’s alpha of 0.91 [55]. The scale consists of 10 items, and was scored on a 4-point Likert scale, with 1 indicating not at all correct and 4 indicating completely correct, with a unidimensional factor structure [56]. The sum of all items is the general self-efficacy score and the total score ranges from 10 to 40, with higher scores indicating higher self-efficacy.

### Statistical analysis

Data analysis was performed using SPSS version 26.0 (IBM SPSS Statistics 26.0, Armonk, NY, USA) and AMOS version 26.0 (SPSS, Chicago, IL, USA). Continuous data were expressed as mean (SD) and categorical data as percentages. Independent samples t-tests or one-way ANOVAs were used to analyze differences in Chineses version of AAUSES scores between sociodemographic categorical and clinical variables, and Bonferroni tests were used to calibrate the test levels for pairwise comparisons. A significance level of *P* < 0.05 was used. The skewness and kurtosis were calculated for each item to determine if the data were normally distributed. When the skewness and kurtosis were between-2 and +2, the data were considered to be normally distributed [57].

### Construct validity

#### Exploratory factor analysis (EFA) and confirmatory factor analysis (CFA)

EFA and CFA were used to examine the construct validity of the Chinese version of AAUSES. The sample of 659 cases was randomly divided into two groups, one group consisted of 331 individuals for EFA, and 328 individuals for CFA.

In the sample 1 (*n *= 331), a principal component analysis (PCA) with Varimax rotation was used to assess the internal structure of the translated the Chinese version of AAUSES. The sample adequacy for the factorability was assessed by the Kaiser-Meyer-Olkin (KMO) [58] metric and Bartlett test of sphericity [59], and sampling was considered adequate when the KMO value was greater than 0.6 and the Bartlett test of sphericity was significant (*P* < 0.05). The factors with eigenvalues > 1 were selected, and the maximum variance orthogonal rotation of the factors was performed. Items with loading values greater than or equal to 0.40 were considered for inclusion in a separate factor [60]. Factors were extracted on the basis of eigenvalues, explained total variance and Scree plot.

In the sample 2 (*n* = 328), CFA was conducted in order to verify the EFA result or test measurement model. CFA can facilitate further evaluation regarding the fitness of the model in line with the structure of the factors [61]. To evaluate the goodness-of-fit of the models, the following indices were evaluated: Chi-square(χ^2^) and degrees of freedom(df), root mean square error of approximation (RMSEA), standardized root mean square residual (SRMR), normed fit index (NFI), goodness of fit index (GFI) and comparative Fit Index (CFI) [62]. A model with χ^2^/df < 3, RMSEA and SRMR < 0.08 [63], and a GFI, CFI and an IFI > 0.90 [64] is considered acceptable.

### Content validity

Content validity index (CVI) was used to evaluate the content validity of the Chinese version of AAUSES. The CVI includes item-level content validity index (I-CVI) and average S-CVI (S-CVI/Ave) [65]. Each expert scored the relevance of each item to the corresponding dimension. A 4-point scale (1 = no relevance, 2 = low relevance, 3 = strong relevance, 4 = very strong relevance) was used to calculate the CVI.

### Discriminant validity and criterion validity

The total the Chinese version of AAUSES scores were sorted from lowest to highest, with the highest 27% of the sample grouped into one group and the lowest 27% into another, and the difference in item scores between the high and low groups was analysed using a two-tailed independent samples t-test. Discriminant validity was considered good if the scores for each item in both groups reached a significant level (*p* < 0.05). We analyzed criterion-related validity by comparing the Chinese version of AAUSES with the GSES scale using Spearman’s correlation.

### Reliability analysis

#### Internal consistency reliability

The internal consistency reliability of the scale was determined by Cronbach alpha coefficient, corrected item- total correlation and retest reliability. Cronbach alpha coefficient equal to or greater than 0.70 is considered acceptable [66]. The corrected item-total correlation, which indicates the correlation of each item with the sum of the other items in the scale, was used at a criterion of 0.3 [67]. Retest reliability reflects the stability of the scale by calculating the retest correlation coefficient (intraclass correlation coefficient, ICC).

#### Test-retest reliability

Two weeks after completing the first response, 30 adults who participated in the first test were recruited to evaluate the test-retest reliability. The correlation between the two tests was assessed using Spearman’s correlation. A correlation coefficient of 0.7 will be used as the recommended threshold [68].

## Results

### Demographics and sample characteristics

In this study, there was a descending order of distribution of the respondents from younger to older age, with the highest percentage among the 18—30 years group (80.48%) and the lowest among those greater than 50 years old who accounted for only 3% of the sample. The majority of the participants were students (58.9%) and the sample was distributed almost equally between place of residence (42.0% live in the city while 58.0% live in the rural), the monthly income with the greatest prevalence among the respondents was less than Ұ10,000 (82.2%). The majority of respondents are females (67.7%), approximately 11.4% of the total number of people with a high school or junior college degree or less, almost 83% have health insurance. Demographic and background information about the sample is summarized in Table 1. The means and standard deviations for all 13 items tested are presented in Table 2, these data were normally distributed according to the skewness and kurtosis figures.

**Table 1:** Demographic characteristics

Variable		Total (N%)
Age (years old)	18–29	526 (79.8)
	30–39	56 (8.5)
	40–49	52 (7.9)
	≥ 50	25 (3.8)
Gender	Male	205 (31.1)
	Female	454 (68.9)
Religious affiliation or not	Yes	43 (6.5)
	No	616 (93.5)
Education level	Junior high school and below	41 (6.2)
	High school or technical secondary school	39 (5.9)
	Junior College or undergraduate	510 (77.4)
	Postgraduate and above	69 (10.5)
Home residence	City	373 (56.6)
	Rural	286 (43.4)
Marital status	Single	371 (56.3)
	In Love	128 (19.4)
	Married	152 (23.1)
	Divorce	6 (0.9)
	Widow	2 (0.3)
Employment Status	Employed	214 (32.5)
	Unemployed	445 (67.5)
Do you have health insurance?	Yes	540 (81.9)
	No	119 (18.1)
Profession	Students	394 (59.8)
	Teachers	50 (7.6)
	Soldiers	4 (0.6)
	Medical practitioner	31 (4.7)
	Farmer	14 (2.1)
	Worker	30 (4.6)
	Housewife	11 (1.7)
	Staff	47 (7.1)
	Individual	15 (2.3)
	Retirement	4 (0.6)
	Others	59 (9.0)
Your family monthly income (yuan)	≤ 5000	282 (42.8)
	5000–10,000	260 (39.5)
	≥ 10,000	117 (17.8)

**Table 2:** Mean (SD) scores with skewness and kurtosis figures (*N* = 659)

Item		Mean(SD)	Skewness	Kurtosis
1	I feel confident I could recover from the cold without taking antibiotics.	67.04 (29.71)	-0.598	-0.472
2	If I were experiencing bronchitis, I feel confident I could try to get better without taking antibiotics.	51.21 (27.21)	-0.226	-0.450
3	I feel confident I could avoid using old/leftover antibiotics when feeling unwell.	59.35 (30.86)	-0.339	-0.760
4	I feel confident I could recover from the flu without taking antibiotics.	58.32 (29.61)	-0.292	-0.685
5	I feel confident I could avoid taking antibiotics prescribed to another person (e.g., family member) when feeling unwell.	58.42 (29.07)	-0.237	-0.670
6	If I had a viral infection, I feel confident I could get better without taking antibiotics.	52.38 (29.27)	-0.076	-0.689
7	I feel confident I could seek an antibiotic prescription from a physician only when necessary.	63.25 (27.07)	-0.365	-0.375
8	I feel confident I could ask my physician any questions about the medication regimen when prescribed antibiotics.	65.05 (26.68)	-0.374	-0.420
9	I feel confident I could avoid taking antibiotics if I had a viral infection.	50.85 (28.45)	-0.064	-0.634
10	I feel confident I could minimize antibiotic use in general.	67.33 (26.03)	-0.416	-0.401
11	I feel confident I could delay seeking physician care for antibiotics until absolutely necessary.	62.91 (26.05)	-0.275	-0.394
12	I feel confident I could trust my physician when he says I do not need to take antibiotics for my illness.	69.64 (25.90)	-0.513	-0.333
13	I feel confident I could delay taking a course of antibiotics until my physician confirms I have a bacterial infection (e.g., wait until the lab-oratory test results come back).	64.87 (25.74)	-0.274	-0.447

### Content validity

The content validity of the Chinese version of AAUSES was evaluated by expert evaluation. The expert group is composed of 6 experts including three psychology experts and three medical experts. The content validity analysis result shows that the I-CVI of the Chinese version of AAUSES is 0.833-1.000, and the S-CVI / Ave is 0.96, which has good content validity.

### Construct validity

#### Exploratory factor analysis (EFA)

**Fig. 1:**
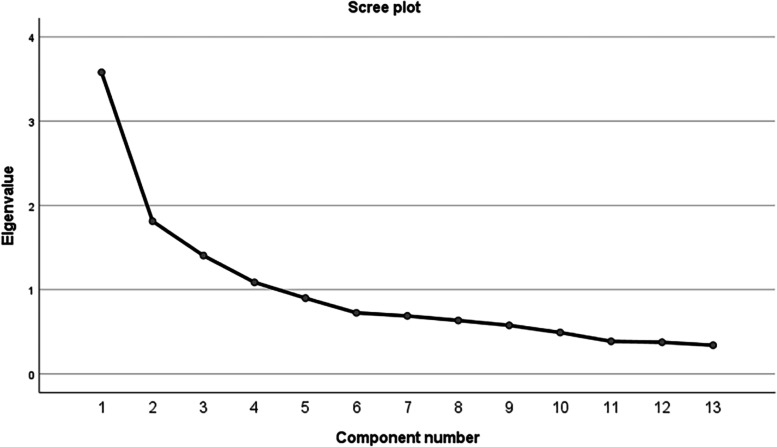
Screen plot of exploratory factor analysis for the Chinese version of the AAUSES (*n* = 331)

Before commencing an EFA, the factorability of the matrix of a sample (*n* = 331) was first examined. The Bartlett test [59] of sphericity was significant (χ^2^78=1050.377; *P* < 0.001), and the KMO index was 0.777, which is greater than the minimum acceptable value of 0.6 [58], suggesting there is sufficient correlation between the variables and the matrix is appropriate for factor extraction. The result showed that four factors had an eigenvalue higher than 1 and yielded 4 common factors with a cumulative variance contribution of 60. 636%, these 4 extracted factors explained 25.08%, 13.32%, 11.21%, 11.02% of the variance, this differs from the 3-factor structural model of the original scale. The factor loadings of the 13 items ranged from 0.520 to 0.862, and all the items were loaded on a single factor, no items were deleted, the results are shown in Table 3. The 4-factor structure was further confirmed by the scree plot, as the descending tendency became weak after the fourth point. The scree plot is shown in Fig. 1.

**Table 3:** Factor loadings of the exploratory factor analysis with 13 items (*n* = 331)

Item number	Factor			
	Factor1	Factor2	Factor3	Factor4
12	0.828			
13	0.745			
10	0.800			
8	0.520			
11	0.735			
7	0.618			
6		0.856		
9		0.862		
2			0.633	
4			0.745	
1			0.635	
5				0.699
3				0.797

#### Confirmatory factor analysis (CFA)

**Fig. 2:**
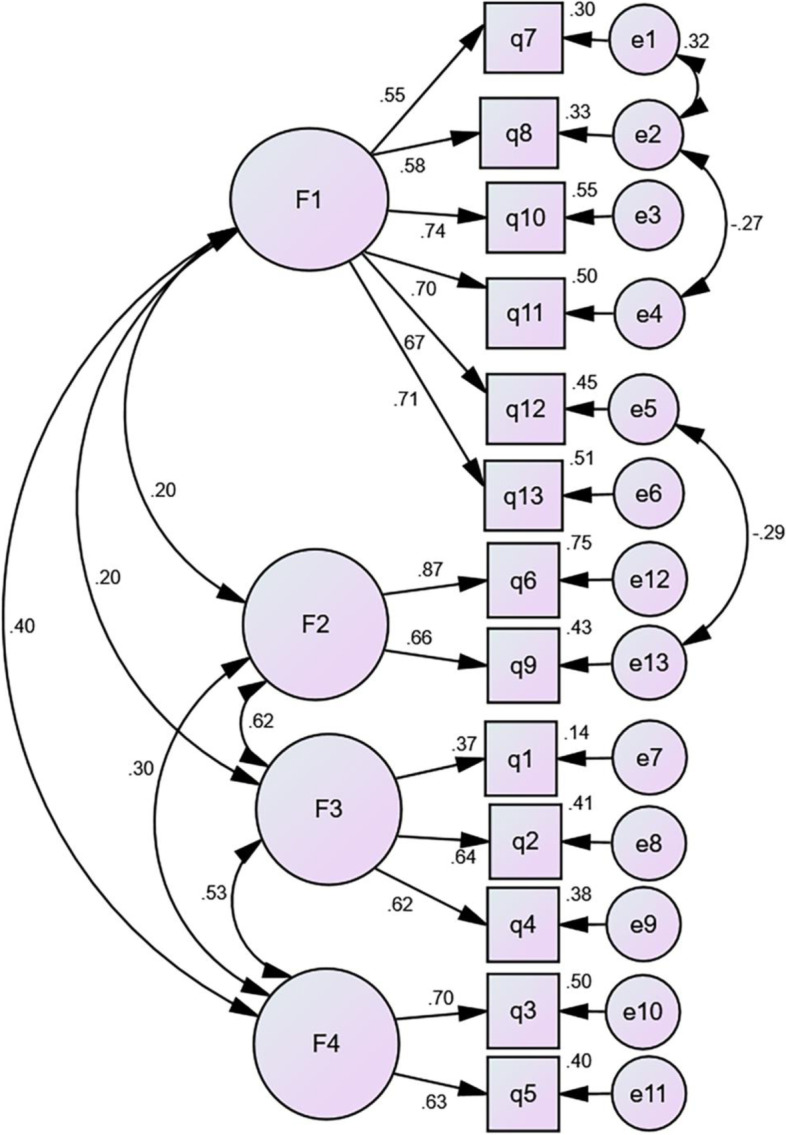
Standardized four-factor structural model of the Chinese version of the AAUSES (*n* = 328). F1 (minimization of antibiotics and trust in physician recommendations, six items), F2 (avoidance of antibiotics for viral infections, two items), F3 (avoidance of taking antibiotics based on previous medication experience, three items), F4 (avoidance of taking old/ other people’s antibiotics, two items)

A CFA was performed on the sample 2 (*n* = 328). In the model fitness index, the chi-square degree of freedom was 2.940, the goodness-of-fit index (GFI) was 0.929, the incremental fit index (IFI) was 0.908, the comparative fit index (CFI) was 0.906, root mean square error of approximation(RMSEA) was 0.077, and standardized root mean residual (SRMR)was 0.0689. The CFA results are shown in Fig. 2.

### Discriminant validity and correlations among factors

#### Discriminant validity

The Chinese version of AAUSES scores of the 659 survey respondents were ranked in order of high and low, and those with scores in the top 27% were grouped into one group and those with scores in the bottom 27% were grouped into another group. After calculation, 650 and 970 scores were selected as thresholds in this study, and those with AAUSES scores below 650 were categorized as the low group and those with scores above 970 were categorized as the high group, and the mean of each item score in the two groups was calculated. Two-tailed independent samples t-test showed there was a significant difference between the items in the two groups (*p*＜0.05). The specific statistical results are shown in Table 4.

**Table 4:** Score comparison between high-score and low-score groups (*N* = 659)

Item	Low-score group (*n* = 251), Mean (SD)	High-score group (*n* = 207), Mean (SD)	t-test(df)	*p*-value
1	39.86﻿ (25.82)	93.89 (9.938)	-28.112 (280.956)	<0.001
2	34.25 (22.05)	70.06 (25.34)	-14.962 (390)	<0.001
3	37.03 (24.69)	83.94 (23.22)	-19.266 (390)	<0.001
4	36.56 (23.01)	85.00 (20.13)	-22.229 (389.655)	<0.001
5	37.36 (22.82)	83.39 (21.77)	-20.323 (390)	<0.001
6	34.81 (20.73)	76.17 (27.08)	-16.743 (331.792)	<0.001
7	40.71 (21.68)	88.22 (18.10)	-23.648 (389.893)	<0.001
8	43.21 (22.90)	87.67 (17.50)	-21.760 (385.897)	<0.001
9	36.23 (21.31)	70.56 (29.31)	-13.057 (320.877)	<0.001
10	43.63 (21.67)	91.67 (12.26)	-27.503 (342.635)	<0.001
11	41.89 (20.15)	87.22 (18.46)	-23.066 (390)	<0.001
12	49.81 (25.33)	91.83 (11.79)	-21.559 (308.759)	<0.001
13	44.48 (21.51)	87.06 (17.23)	-21.363 (390)	<0.001

### Correlations among factors

The correlation analysis results (Table 5) showed that the Chinese version of AAUSES had a positive correlation between the total score and each dimension, and each dimension score and total score are positively correlated with the GSES score.

**Table 5:** Pearson’s correlations between the Chinese version of AAUSES and subscales and GSES

	AAUSES	Factor 1	Factor 2	Factor 3	Factor 4
Factor 1	0.875^**^	-	-	-	-
Factor 2	0.770^**^	0.595^**^	-	-	-
Factor 3	0.853^**^	0.594^**^	0.374^**^	-	
Factor 4	0.656^**^	0.432^**^	0.405^**^	0.516^**^	-
GSES	0.302^**^	0.278^**^	0.195^**^	0.249^**^	0.246^**^

^**^Significant correlation at the 0.01 level (two-sided)

-Not available

### Reliability analysis

#### Internal consistency reliability

Reliability analysis results showed that the Chinese version of AAUSES had ideal internal consistency, with the overall Cronbach alpha coefficient being 0.910 and Cronbach alpha coefficients for the 4 factors being 0.911, 0.707, 0.742, and 0.939, all were greater than the minimum acceptable value of Cronbach alpha coefficient [66]. Table 6 showed the correlation coefficient between the 13 items of the questionnaire and the total score, and presented the Cronbach alpha coefficients after removing an item from the questionnaire, all of which were lower than the Cronbach alpha coefficient of 0.90 before the removal. In addition, the corrected item-total correlations for the items ranged from 0.488 to 0.736, all of which were well above 0.3 [67]. Therefore, all 13 items were retained and none were deleted.

#### Test-retest reliability

Two weeks later, a random sample of 30 adults who participated in the first survey completed the questionnaire again and the Spearman correlation coefficient was 0.947, which was greater than 0.7, and the Chinese version of the AAUSES scale had good test-retest reliability.

**Table 6:** Correlation between each item of the questionnaire and the total score (*N* = 659)

	Cronbach alpha if the item was deleted	r	Corrected item-total correlation
1	0.902	0.778	0.656
2	0.909	0.578	0.488
3	0.906	0.659	0.576
4	0.903	0.713	0.633
5	0.904	0.698	0.629
6	0.907	0.622	0.548
7	0.900	0.756	0.711
8	0.902	0.709	0.660
9	0.909	0.577	0.507
10	0.899	0.762	0.736
11	0.900	0.747	0.724
12	0.902	0.695	0.662
13	0.901	0.696	0.701

### Analysis of differences in chinese version of AAUSES with different sociodemographic information

Among the general demographic variables, there are statistically significant differences in the scores of the Chinese version of AAUSES among different education levels, occupations, per capita monthly income, family location, and whether there is medical insurance. Among the clinical variables, there were no differences in the total scores for whether or not they had taken antibiotics and the number of times they had taken antibiotics when they had a cold. However, those who had taken antibiotics for a cold or flu scored significantly lower than those who had not, and those who were very concerned about antibiotic resistance scored higher than those who had never heard of antibiotic resistance and those who were less concerned about antibiotic resistance people. The specific results are shown in Table 7.

**Table 7:** Comparison of the Chinese version of the AAUSES of subjects with different characteristics

Variable		Mean (SD)	t/F	*p*-value	Pairwise differences
Age group (years)	18-29	61.01 (18.61)	0.732	0.570	
	30-39	58.65 (21.27)			
	40-49	61.68 (24.35)			
	≥50	63.85 (23.39)			
Gender	Male	61.04 (20.68)	0.045	0.964	
	Female	60.96 (19.14)			
Religious affiliation or not	Yes	63.77 (18.74)	0.965	0.335	
	No	60.79 (19.68)			
Education level	Junior high school and below (1)	51.28 (25.57)	6.208	**<0.001**	(4)(3)>(1)(2)
	High school or technical secondary school (2)	54.60 (18.97)			
	Junior College or undergraduate (3)	61.66 (18.73)			
	Postgraduate and above (4)	65.34 (20.21)			
Home residence	City (1)	63.01 (19.41)	3.051	**0.002**	(1)>(2)
	Rural (2)	58.34 (19.60)			
Marital status	Single	60.73 (19.10)	0.845	0.897	
	In Love	62.33 (18.05)			
	Married	60.61 (21.84)			
	Divorce	52.18 (23.61)			
	Widow	77.70 (28.28)			
Employment Status	Employed	59.60 (20.54)	-1.252	0.211	
	Unemployed	61.65 (19.14)			
Do you have health insurance?	Yes	61.71 (19.83)	2.032	**0.043**	
	No	57.69 (18.33)			
Profession	Students (1)	62.43 (18.40)	3.253	**<0.001**	(1) (2) (4 (6) (7) (8) (10)>(5) (1) (2) (4 (6) (8) (10)>(11)
	Teachers (2)	60.55 (21.17)			
	Soldiers (3)	51.15 (12.71)			
	Medical practitioner (4)	64.34 (21.55)			
	Farmer (5)	46.48 (21.73)			
	Worker (6)	60.36 (20.62)			
	Housewife (7)	62.80 (20.10)			
	Staff (8)	64.29 (15.30)			
	Individual (9)	58.98 (20.60)			
	Retirement (10)	76.15 (23.07)			
	Others (11)	50.87 (23.09)			
Your family monthly income (yuan)	≤5000	57.24 (19.97)	11.173	**<0.001**	(2)(3)>(1)
	5000-10000	62.49 (19.41)			
	≥10000	66.68 (17.44)			
Taking antibiotics or not	Yes	60.64 (19.90)	-0.576	0.565	
	No	61.55 (19.17)			
Whether to take antibiotics to treat colds or flu	Yes	59.83 (19.17)	-1.785	0.061	
	No	62.75 (20.18)			
Number of times a cold or flu is treated with antibiotics	Never(1)	62.02 (20.80)	1.668	0.556	
	Once (2)	61.20 (15.64)			
	Twice (3)	64.44 (17.80)			
	Three times (4)	55.09 (21.06)			
	More than three times (5)	59.43 (19.85)			
Have you listened to antibiotic resistance?	Yes	62.33 (19.39)	3.779	**<0.001**	
	No	54.90 (19.57)			
You are concerned about antibiotic resistance	Didn't hear antibiotic resistance (1)	50.50 (19.28)	10.582	**<0.001**	(2)(3)(4)>(1) (2)>(3)(4)
	Very much agree (2)	68.29 (19.84)			
	A little agreed (3)	61.10 (18.52)			
	Uncertain (4)	57.65 (18.18)			
	A little disagree (5)	61.07 (19.01)			
	Strongly disagree (6)	65.13 (22.63)			

## Discussion

To our knowledge, this study was the first attempt to introduce the scale to measure the appropriate antibiotic use self-efficacy of adults. We translated the scale into Chinese after a rigorous cultural adaptation process and validated a measure with adequate internal consistency, test-retest reliability, content validity, construct validity, and discriminant validity, which is especially suitable to evaluate the appropriate antibiotic use self-efficacy of adults. Finally, a Chinese version of the scale with 13 items and a four-factor structure was developed.

### The chinese version of AAUSES has good reliability

Reliability analysis reflects the stability of the structure of the scale being measured [69]. The reliability of the AAUSES was assessed using the Cronbach alpha coefficient, item-total correlations, and test–retest. In this study, the Cronbach alpha coefficient of the Chinese version of the AAUSES in this study was 0.910, which demonstrated adequate stability of AAUSES measures of individual self-efficacy for rational antibiotic use. The item-total score correlation coefficients were all above 0.30, which confirmed the Chinese version of AAUSES had good internal consistency. In addition, the ICC for the scale was 0.947 in the test–retest study. The results indicated that the Chinese version of AAUSES had stable repeatability.

### The chinese version of AAUSES has good validity

Validity refers to the extent to which the measured tool accurately corresponds to the real world [23]. We assessed the content validity, discriminant validity and construct validity of the scale. If the S-CVI/Ave is above 0.90, it is considered to have good content validity. The content validity of the Chinese version of AAUSES is 0.96, which has good content validity [70]. The discriminant validity results showed that the score of each item in the 2 groups reached the level of significance (*P* < 0.05), which was considered good. It is generally accepted that the ideal structural validity should be such that (1) the factors extracted through exploratory analysis explain more than 40.00% of the total variance, and (2) each item has a loading value higher than 0.4 on a single factor and lower loading values on the other factors [71]. In this study, four factors were extracted through exploratory factor analysis, explaining 60.636% of the variance in the total data. The factor loadings of the 13 items ranged from 0.520 to 0.862. Moreover, the CFA also showed that the individual Chinese version of AAUSES projects fit well with this four-dimensional structural model, as the model fit indices all meet acceptable criteria. Overall, the Chinese version of the AAUSES showed optimal validity among Chinese adults.

### There is a reasonable explanation for the addition of a new dimension

This study determined finally that the scale is a four-dimensional structure (minimization of antibiotics and trust in physician recommendations (including items 7,8,10,11,12,13), avoidance of taking antibiotics based on previous medication experience(including items 1,2,4), avoidance of antibiotics for viral infections(including items 6,9), taking old/other people’s antibiotics(including items 3,5)). This differed from the three dimensional structure of the original version(minimization of antibiotics and trust in physician recommendations(including items 7,8,10,11,12,13), avoidance of antibiotics for viral infections(including items1,2,4,6,9), taking old/other people’s antibiotics(including items 3,5)) [50]. In this study, the dimension of the original scale avoidance of antibiotics for viral infections (including items 1, 2, 4, 6, 9) was split into two dimensions. Based on expert opinions, literature review, and the underlying characteristics of the items, we renamed it avoidance of taking antibiotics based on previous medication experience (including items1, 2, 4) and avoidance of antibiotics for viral infections(including items 6,9), which proved to be more suitable for Chinese people. There are relatively reasonable explanations for item1, item2 and item4 on a common dimension. Firstly, during the translation process, cross-cultural adaptations have been made to items that do not conform to Chinese expression habits, which affected the original structure of the scale to some extent. Secondly, the use of antibiotics differs between domestic and foreign countries, it may be attributable to the differences in socio-economic and sample population. Thirdly, Bandura identified four main sources of self-efficacy beliefs: active mastery experiences, alternative experiences, verbal persuasion, and physiological responses. Mastery experiences are considered to be the most influential source of self-efficacy [72–74]. Active mastery experiences are interpreted as successful outcomes increasing self-efficacy and failed experiences decreasing self-efficacy. However, there is a low self-efficacy for rational antibiotic use due to the misconception of “previous medication experience”. Most people lack an understanding of the natural course of self-limiting illnesses and therefore assume that antibiotics are effective in treating viral infections based on previous experience with medication [75]. Thus most customers with symptoms of colds, flu, bronchitis and respiratory infections (cough, runny nose, sore throat) go to pharmacies for antibiotics, yet these symptoms are usually viral [76]. A survey by Joran showed that participants who self-administered antibiotics mainly used them for sore throats and influenza, with 35. 2% based on their previous experience with antibiotics [77]. Therefore, we named this dimension avoidance of taking antibiotics based on previous medication experience.

### Comparison of the original AAUSES and the chinese version of the AAUSES self-efficacy score

In this study, the self-efficacy score was much lower than the self-efficacy score for antibiotic use on the original scale [50]. This may be related to socioeconomic differences and different sample populations. According to Levy and Marshall [78], in developing countries, antibiotics may be at greater risk of resistance. Because they are cheap and readily available and often used unnecessarily. In China, the Chinese have less knowledge about the rational use of antibiotics and are unaware that irrational antibiotic use can lead to antibiotic resistance, and misconceptions about antibiotic use are evident in the general population [79]. The results of a study showed that about half of the population did not know that antibiotics do not treat colds or that they are not effective against viral infections [80]. Compared with countries such as the United States and the United Kingdom, most efforts in China have focused on stewardship initiatives and regulations, training, and support for clinicians, while education efforts to improve antibiotic knowledge and promote the rational use of antibiotics have lagged relatively behind [81].

### Analysis of differences in self-efficacy of general information on the rational use of antibiotics

We found that individuals with higher levels of education have higher self-efficacy scores for the rational use of antibiotics, the more literate and educated participants clearly understood the dosage and side effects of antibiotics and are more confident in their rational use of antibiotics. The results of a study in Nepal also showed that better educated respondents had a better understanding of antibiotics and had more appropriate attitudes and practices [82]. We also found there were statistically significant differences between different occupations, with medical professionals having the highest scores and farmers having the lowest self-efficacy scores, This is consistent with a study investigating the Knowledge, Attitudes and Practice (KAP), which found that healthcare practitioners significantly outperformed non-practitioner group practice scores [83]. However, a study in eastern Turkey found that farmers were far less aware of antibiotic use, duration, storage, and resistance than expected [84]. There were statistically significant differences by household location, with urban households scoring significantly higher than rural households. Urban areas have a faster development of economic level than rural areas, richer sources of information, and urban residents are more likely to acquire antibiotic-related knowledge. A study in India showed that the prevalence of self-medication in rural populations was higher than in urban areas and the knowledge level of urban residents is higher than that of rural residents [85]. There were statistically significant differences in health insurance status. A study finds significant differences in residents’ attitudes towards antibiotic use and whether they have medical insurance [86]. Those who had heard of antibiotic resistance and those who were worried about antibiotic resistance had higher self-efficacy scores than those who had not heard of antibiotic resistance. Researchers found that respondents familiar with antibiotic-related knowledge are more likely to have a positive attitude towards antibiotic use and vice versa [87]. This study also found that self-efficacy scores for rational antibiotic use were related to income, with higher income being associated with higher self-self-efficacy scores, which may be related to the fact that participants with higher income levels were less likely to self-treat [88, 89]. A study by Allcock et al. [90] showed that people with lower incomes more often go to pharmacies to buy antibiotics because they are cheap and readily available over the counter. This suggests that it is essential and urgent to inform the general population about the rational use of antibiotics and its consequences in less economically developed areas, especially in rural areas and among less educated people. Future systematic and in-depth research should be conducted on the factors that influence the self-efficacy of rational antibiotic use to provide a scientific basis for antibiotic managers to improve the self-efficacy of rational antibiotic use in adults.

## Limitations

To the best of our knowledge, this is the first study to translate and validate AAUSES in China, and some limitations should be acknowledged. Firstly, the majority of the study participants recruited for this study using convenience sampling were young and highly educated, so this may overestimate the level of self-efficacy scores for rational antibiotic use among Chinese adults and should therefore be validated in a broader population in the future. Secondly, as with most cross-sectional studies, there are inherent drawbacks such as recall bias and difficulty in inferring causal conclusions. Thirdly, this paper is the first study to localize the AAUSES, and thus the reliability and validity of the scale need to be further tested through future studies.

## Conclusions

The Chinese version of the AAUSES, consisting of 13 items, and supporting a four-factor structure, demonstrated good validity and reliability in this study and proved to be a valid tool for assessing self-efficacy for appropriate antibiotic use among Chinese adults. In addition, future research should examine the psychometric properties of the Chinese version of AAUSES in a broader sample of Chinese adults, particularly in areas where the irrational use of antibiotics is more prevalent. We believe that there is a need to use the Chinese version of the AAUSES to assess the self-efficacy of Chinese adults in the rational use of antibiotics in future studies. In addition, future research should pay more attention to the relevant factors affecting the self-efficacy of rational use of antibiotics, this will provide a basis for public health authorities to develop policy guidance and interventions to improve public self-efficacy in the rational use of antibiotics, promote changes in irrational behavior in antibiotic use, and reduce the health hazards and economic losses caused by antibiotic resistance.

## Data Availability

The datasets generated and/or analysed during the current study are not publicly available due Chinese people are relatively secretive about their lives and thoughts, although informed consent was obtained from study subjects prior to the survey and the findings were largely reported but are available from the corresponding author on reasonable request.“

## Abbreviations

AMR: Anti-Microbial Resistance
AAUSES: The Appropriate Antibiotic Use Self-Efficacy Scale
GSES: The General Self-Efficacy Scale
PCA: Principal components analysis
EFA: Exploratory Factor Analysis
CFA: Confirmatory Factor Analysis
CFI: Comparative Fit Index
TLI: Tucker-Lewis Index
RMSEA: Root Mean Square Error of Approximation
SRMR: Standardized Root Mean Square Residual

## References

[1] Sengupta S, Chattopadhyay MK, Grossart HP (2013). The multifaceted roles of antibiotics and antibiotic resistance in nature. Front Microbiol.

[2] Sutherland ME (2018). Antibiotic use across the globe. Nat Human Behav.

[3] ECDC E (2009). The bacterial challenge–time to react a call to narrow the gap between multidrug-resistant bacteria in the eu and development of new antibacterial agents.

[4] O’Neill J (2021). Antimicrobial Resistance: Tackling a crisis for the health and wealth of nations. The Review on Antimicrobial Resistance. December 2014. Review on Antimicrobial Resistance.

[5] Arason VA, Sigurdsson JA, Erlendsdottir H, Gudmundsson S, Kristinsson KG (2006). The role of antimicrobial use in the epidemiology of resistant pneumococci: a 10-year follow up. Microbial Drug Resist.

[6] Bronzwaer SL, Cars O, Buchholz U, Mölstad S, Goettsch W, Veldhuijzen IK, Kool JL, Sprenger MJ, Degener JE (2002). The relationship between antimicrobial use and antimicrobial resistance in Europe. Emerg Infect Dis.

[7] Goossens H, Ferech M, Vander Stichele R, Elseviers M, Group EP (2005). Outpatient antibiotic use in Europe and association with resistance: a cross-national database study. Lancet.

[8] Mangione-Smith R, McGlynn EA, Elliott MN, Krogstad P, Brook RH (1999). The relationship between perceived parental expectations and pediatrician antimicrobial prescribing behavior. Pediatrics.

[9] Auta A, Hadi MA, Oga E, Adewuyi EO, Abdu-Aguye SN, Adeloye D, Strickland-Hodge B, Morgan DJ (2019). Global access to antibiotics without prescription in community pharmacies: a systematic review and meta-analysis. J Infect.

[10] Nepal G, Bhatta S: Self-medication with antibiotics in WHO Southeast Asian Region: a systematic review. Cureus 2018, 10(4). 10.7759/cureus.2428 10.7759/cureus.2428 PMC5988199 29876150

[11] Kianmehr H, Sabounchi NS, Seyedzadeh Sabounchi S, Cosler LE (2019). Patient expectation trends on receiving antibiotic prescriptions for respiratory tract infections: A systematic review and meta-regression analysis. Int J Clin Pract.

[12] Hu Y, Wang X, Tucker JD, Little P, Moore M, Fukuda K, Zhou X (2018). Knowledge, attitude, and practice with respect to antibiotic use among Chinese medical students: a multicentre cross-sectional study. Int J Environ Res Public Health.

[13] Wang X, Lin L, Xuan Z, Li L, Zhou X (2018). Keeping antibiotics at home promotes self-medication with antibiotics among Chinese university students. Int J Environ Res Public Health.

[14] Aljayyousi GF, Abdel-Rahman ME, El-Heneidy A, Kurdi R, Faisal E (2019). Public practices on antibiotic use: A cross-sectional study among Qatar University students and their family members. PloS one.

[15] Grigoryan L, Burgerhof JG, Haaijer-Ruskamp FM, Degener JE, Deschepper R, Monnet DL, Di Matteo A, Scicluna EA, Bara A-C, Lundborg CS (2007). Is self-medication with antibiotics in Europe driven by prescribed use?. J Antimicrobial Chemother.

[16] Lv B, Zhou Z, Xu G, Yang D, Wu L, Shen Q, Jiang M, Wang X, Zhao G, Yang S (2014). Knowledge, attitudes and practices concerning self-medication with antibiotics among university students in western China. Trop Med Int Health.

[17] Liu C, Liu C, Wang D, Deng Z, Tang Y, Zhang X (2019). Determinants of antibiotic prescribing behaviors of primary care physicians in Hubei of China: a structural equation model based on the theory of planned behavior. Antimicrob Resist Infect Control.

[18] Watkins LKF, Sanchez GV, Albert AP, Roberts RM, Hicks LA (2015). Knowledge and attitudes regarding antibiotic use among adult consumers, adult Hispanic consumers, and health care providers—United States, 2012–2013. MMWR Morb Mortal Wkly Rep.

[19] Zhang Q-Q, Ying G-G, Pan C-G, Liu Y-S, Zhao J-L (2015). Comprehensive evaluation of antibiotics emission and fate in the river basins of China: source analysis, multimedia modeling, and linkage to bacterial resistance. Environ Sci Technol.

[20] Xiao Y, Zhang J, Zheng B, Zhao L, Li S, Li L (2013). Changes in Chinese policies to promote the rational use of antibiotics. PLoS medicine.

[21] Wang X, Xuan Z, Storella TH, Zhou X (2020). Determinants of non-prescription antibiotic dispensing in Chinese community pharmacies from socio-ecological and health system perspectives. Soc Sci Med.

[22] Fang Y: China should curb non-prescription use of antibiotics in the community. BMJ 2014, 348. 10.1136/bmj.g4233 10.1136/bmj.g4233 24982508

[23] Cheng J, Coope C, Chai J, Oliver I, Kessel A, Wang D, Sun Y (2018). Knowledge and behaviors in relation to antibiotic use among rural residents in Anhui China. Pharmacoepidemiol Drug Saf.

[24] Wang X, Peng D, Wang W, Xu Y, Zhou X, Hesketh T (2017). Massive misuse of antibiotics by university students in all regions of China: implications for national policy. Int J Antimicrobial Agents.

[25] Saleh N, Awada S, Awwad R, Jibai S, Arfoul C, Zaiter L, Dib W, Salameh P (2015). Evaluation of antibiotic prescription in the Lebanese community: a pilot study. Infect Ecol Epidemiol.

[26] Farah R, Lahoud N, Salameh P, Saleh N (2015). Antibiotic dispensation by Lebanese pharmacists: a comparison of higher and lower socio-economic levels. J Infect Public Health.

[27] Cheaito L, Azizi S, Saleh N, Salameh P (2014). Assessment of self-medication in population buying antibiotics in pharmacies: a pilot study from Beirut and its suburbs. Int J Public Health.

[28] Mouhieddine TH, Olleik Z, Itani MM, Kawtharani S, Nassar H, Hassoun R, Houmani Z, El Zein Z, Fakih R, Mortada IK (2015). Assessing the Lebanese population for their knowledge, attitudes and practices of antibiotic usage. J Infect Public Health.

[29] Gunasekera YD, Kinnison T, Kottawatta SA, Silva-Fletcher A, Kalupahana RS: Misconceptions of Antibiotics as a Potential Explanation for Their Misuse. A Survey of the General Public in a Rural and Urban Community in Sri Lanka. Antibiotics (Basel) 2022, 11(4). 10.3390/antibiotics11040454 10.3390/antibiotics11040454 PMC9024968 35453206

[30] Paredes JL, Navarro R, Watanabe T, Morán F, Balmaceda MP, Reateguí A, Elias R, Bardellini M, Ochoa TJ (2022). Knowledge, attitudes and practices of parents towards antibiotic use in rural communities in Peru: a cross-sectional multicentre study. BMC Public Health.

[31] Gualano MR, Gili R, Scaioli G, Bert F, Siliquini R (2015). General population’s knowledge and attitudes about antibiotics: a systematic review and meta-analysis. Pharmacoepidemiol Drug Saf.

[32] Boiko O, Gulliford MC, Burgess C (2020). Revisiting patient expectations and experiences of antibiotics in an era of antimicrobial resistance: Qualitative study. Health Expect.

[33] Gaarslev C, Yee M, Chan G, Fletcher-Lartey S, Khan R (2016). A mixed methods study to understand patient expectations for antibiotics for an upper respiratory tract infection. Antimicrob Resist Infect Control.

[34] Horvat O, Tomas A, Paut Kusturica M, Bukumiric D, Blagojevic B, Kovacevic Z (2020). Serbian students’ knowledge, attitudes and behaviour towards antibiotic use: is there room for improvement?. Int J Public Health.

[35] Hu Y, Wang X, Tucker JD, Little P, Moore M, Fukuda K, Zhou X. Knowledge, Attitude, and Practice with Respect to Antibiotic Use among Chinese Medical Students: A Multicentre Cross-Sectional Study. Int J Environ Res Public Health. 2018;15(6). 10.3390/ijerph15061165 10.3390/ijerph15061165 PMC6025109 29867005

[36] Lum EPM, Page K, Nissen L, Doust J, Graves N (2017). Australian consumer perspectives, attitudes and behaviours on antibiotic use and antibiotic resistance: a qualitative study with implications for public health policy and practice. BMC Public Health.

[37] Hawkins O, Scott AM, Montgomery A, Nicholas B, Mullan J, van Oijen A, Degeling C (2022). Comparing public attitudes, knowledge, beliefs and behaviours towards antibiotics and antimicrobial resistance in Australia, United Kingdom, and Sweden (2010–2021): A systematic review, meta-analysis, and comparative policy analysis. PLoS One.

[38] Strecher VJ, DeVellis BM, Becker MH, Rosenstock IM (1986). The role of self-efficacy in achieving health behavior change. Health Educ Q.

[39] Brouwer-Goossensen D, van Genugten L, Lingsma HF, Dippel DW, Koudstaal PJ, den Hertog HM (2018). Self-efficacy for health-related behaviour change in patients with TIA or minor ischemic stroke. Psychology & Health.

[40] Hyde J, Hankins M, Deale A, Marteau TM (2008). Interventions to increase self-efficacy in the context of addiction behaviours: a systematic literature review. J Health Psychol.

[41] Bandura A (1977). Self-efficacy: toward a unifying theory of behavioral change. Psychol Rev.

[42] Bandura A (1977). Self-efficacy: toward a unifying theory of behavioral change. Psychol Rev.

[43] Cao Y, Chen W, Zhang S, Jiang H, Liu H, Hua Z, Ren D, Ren J (2019). Development And Preliminary Evaluation Of Psychometric Properties Of A Tuberculosis Self-Efficacy Scale (TBSES). Patient Prefer Adherence.

[44] Huang YM, Shiyanbola OO, Smith PD (2018). Association of health literacy and medication self-efficacy with medication adherence and diabetes control. Patient Prefer Adherence.

[45] Du C, Wu S, Liu H, Hu Y, Li J (2018). Correlation of long-term medication behaviour self-efficacy with social support and medication knowledge of kidney transplant recipients. Int J Nurs Sci.

[46] Risser J, Jacobson TA, Kripalani S (2007). Development and psychometric evaluation of the Self-efficacy for Appropriate Medication Use Scale (SEAMS) in low-literacy patients with chronic disease. J Nurs Meas.

[47] Ogedegbe G, Mancuso CA, Allegrante JP, Charlson ME (2003). Development and evaluation of a medication adherence self-efficacy scale in hypertensive African-American patients. J Clin Epidemiol.

[48] Erlen JA, Cha ES, Kim KH, Caruthers D, Sereika SM (2010). The HIV Medication Taking Self-efficacy Scale: psychometric evaluation. J Adv Nurs.

[49] Sleath B, Carpenter DM, Blalock SJ, Davis SA, Hickson RP, Lee C, Ferreri SP, Scott JE, Rodebaugh LB, Cummings DM (2016). Development of a new diabetes medication self-efficacy scale and its association with both reported problems in using diabetes medications and self-reported adherence. Patient Prefer Adherence.

[50] Hill EM, Watkins K (2018). Development and initial validation of the appropriate antibiotic use self-efficacy scale. Patient Educ Couns.

[51] Costello AB, Osborne J (2005). Best practices in exploratory factor analysis: Four recommendations for getting the most from your analysis. Pract Assess Res Eval.

[52] Association WM (2001). World Medical Association Declaration of Helsinki. Ethical principles for medical research involving human subjects. Bull World Health Organization.

[53] Brislin RW (1970). Back-translation for cross-cultural research. J Cross-cultural Psychol.

[54] Schwarzer R, Bäßler J, Kwiatek P, Schröder K, Zhang JX (1997). The assessment of optimistic self-beliefs: comparison of the German, Spanish, and Chinese versions of the general self‐efficacy scale. Appl Psychol.

[55] Schwarzer R, Bäßler J, Kwiatek P, Schröder K, Zhang JX. The assessment of optimistic self‐beliefs: comparison of the German, Spanish, and Chinese versions of the general self‐efficacy scale. Appl Psychol. 1997;46(1):69–88. 10.1111/j.1464-0597.1997.tb01096.x

[56] Cheung S-K, Sun SY (1999). Assessment of optimistic self-beliefs: further validation of the Chinese version of the General Self-Efficacy Scale. Psychol Rep.

[57] Adawi M, Bragazzi NL, Argumosa-Villar L, Boada-Grau J, Vigil-Colet A, Yildirim C, Del Puente G, Watad A (2018). Translation and validation of the Nomophobia Questionnaire in the Italian language: Exploratory factor analysis. JMIR mHealth uHealth.

[58] Kaiser HF (1974). An index of factorial simplicity. Psychometrika.

[59] Bartlett MS (1954). A Note on the Multiplying Factors for Various χ2 Approximations. J Royal Stat Soc Series B (Methodological).

[60] Iacobucci D, Ruvio A, Román S, Moon S, Herr PM (2022). How many factors in factor analysis? New insights about parallel analysis with confidence intervals. J Bus Res.

[61] Hair JF, Black WC, Babin BJ (2010). RE Anderson Multivariate data analysis: a global perspective.

[62] McCoach  DB, Gable  RK, Madura  JP (2013). Instrument development in the affective domain.

[63] Edwards  ED, Brown Timothy A (2010). Book Review. Confirmatory factor analysis for applied research.

[64] Lt Hu (1999). Bentler PM: Cutoff criteria for fit indexes in covariance structure analysis: conventional criteria versus new alternatives. Structural Equation Model A Multidisciplinary J.

[65] Lynn MR (1986). Determination and quantification of content validity. Nurs Res..

[66] Cronbach  LJ (1951). Coefficient alpha and the internal structure of tests. Psychometrika.

[67] Ferketich S (1991). Focus on psychometrics. Aspects of item analysis. Res Nurs Health.

[68] DeVon HA, Block ME, Moyle-Wright P, Ernst DM, Hayden SJ, Lazzara DJ, Savoy SM, Kostas-Polston E (2007). A psychometric toolbox for testing validity and reliability. J Nurs Scholarsh.

[69] Koo TK, Li MY (2016). A guideline of selecting and reporting intraclass correlation coefficients for reliability research. J Chiropr Med.

[70] Polit DF, Beck CT (2006). The content validity index: are you sure you know what’s being reported? Critique and recommendations. Res Nurs Health.

[71] Boone KB, Pontón MO, Gorsuch RL, González JJ, Miller BL (1998). Factor analysis of four measures of prefrontal lobe functioning. Arch Clin Neuropsychol.

[72] Lightsey R. Albert Bandura and the exercise of self-efficacy. J Cogn Psychother. 1999;13(2):158.

[73] Pajares F (1997). Current directions in self-efficacy research. Adv Motiv Achiev.

[74] Bong M, Skaalvik EM (2003). Academic self-concept and self-efficacy: How different are they really?. Educ Psychol Rev.

[75] Butler CC, Rollnick S, Pill R, Maggs-Rapport F, Stott N (1998). Understanding the culture of prescribing: qualitative study of general practitioners’ and patients’ perceptions of antibiotics for sore throats. BMJ.

[76] Nguyen NV, Do NTT, Nguyen CTK, Tran TK, Ho PD, Nguyen HH, Vu HTL, Wertheim HF, van Doorn HR, Lewycka S (2020). Community-level consumption of antibiotics according to the AWaRe (Access, Watch, Reserve) classification in rural Vietnam. JAC Antimicrob Resist.

[77] Nusair MB, Al-Azzam S, Alhamad H, Momani MY (2021). The prevalence and patterns of self-medication with antibiotics in Jordan: A community-based study. Int J Clin Pract.

[78] Levy SB, Marshall B (2004). Antibacterial resistance worldwide: causes, challenges and responses. Nat Med.

[79] Organization WH (2015). Antibiotic resistance: Multi-country public awareness survey.

[80] Gualano MR, Gili R, Scaioli G, Bert F, Siliquini R (2015). General population’s knowledge and attitudes about antibiotics: a systematic review and meta-analysis. Pharmacoepidemiol Drug Saf.

[81] Liang X, Xia T, Zhang X, Jin C (2014). Governance structure reform and antibiotics prescription in community health centres in Shenzhen, China. Fam Pract.

[82] Mouhieddine TH, Olleik Z, Itani MM, Kawtharani S, Nassar H, Hassoun R, Houmani Z, El Zein Z, Fakih R, Mortada IK (2015). Assessing the Lebanese population for their knowledge, attitudes and practices of antibiotic usage. J Infect Public Health.

[83] Firouzabadi D, Mahmoudi L (2020). Knowledge, attitude, and practice of health care workers towards antibiotic resistance and antimicrobial stewardship programmes: a cross-sectional study. J Eval Clin Pract.

[84] Ozturk Y, Celik S, Sahin E, Acik MN, Cetinkaya B: Assessment of Farmers’ Knowledge, Attitudes and Practices on Antibiotics and Antimicrobial Resistance. Animals (Basel) 2019, 9(9). 10.3390/ani9090653 10.3390/ani9090653 PMC6770244 31487911

[85] Singh NK, Trivedi N, Elnour AA, Patel I (2015). Evaluation of knowledge, attitude and practice about self-medication among rural and urban north Indian population. Age.

[86] Altorkmani A, Alzabibi MA, Shibani M, Ismail H, Sawaf B, Daher N, Al-Moujahed A (2021). Assessing the Syrian population’s knowledge, attitudes, and practices regarding antibiotic usage. Avicenna J Med.

[87] Abdelrahman T, Alsaeed M, Karam R, Alkhthami A, Alswat O, Alzahrani A, Hendi O, Jawad H (2017). Misuse of antibiotics and antibiotic resistance: a public population-based health survey in Altaif-Saudi Arabia. WJPMR.

[88] Sahoo KC, Tamhankar AJ, Johansson E, Stålsby Lundborg C (2014). Community perceptions of infectious diseases, antibiotic use and antibiotic resistance in context of environmental changes: a study in Odisha, India. Health Expect.

[89] Vallin M, Polyzoi M, Marrone G, Rosales-Klintz S, Tegmark Wisell K, Stålsby Lundborg C (2016). Knowledge and attitudes towards antibiotic use and resistance - a latent class analysis of a Swedish population-based sample. PLoS One.

[90] Allcock S, Young EH, Holmes M, Gurdasani D, Dougan G, Sandhu MS, Solomon L, Török ME (2017). Erratum: Antimicrobial resistance in human populations: challenges and opportunities - ERRATUM. Glob Health Epidemiol Genom.

